# ATP-binding cassette (ABC) import systems of *Mycobacterium tuberculosis*: target for drug and vaccine development

**DOI:** 10.1080/22221751.2020.1714488

**Published:** 2020-01-27

**Authors:** Dharmendra Kumar Soni, Suresh Kumar Dubey, Rakesh Bhatnagar

**Affiliations:** a Molecular Biology and Genetic Engineering Laboratory, School of Biotechnology, Jawaharlal Nehru University, New Delhi, India; b Department of Botany, Institute of Science, Banaras Hindu University, Varanasi, India

**Keywords:** *Mycobacterium tuberculosis*, ABC importers, pathogenesis, inhibitor, drug, vaccine

## Abstract

Nutrient procurement specifically from nutrient-limiting environment is essential for pathogenic bacteria to survive and/or persist within the host. Long-term survival or persistent infection is one of the main reasons for the overuse of antibiotics, and contributes to the development and spread of antibiotic resistance. *Mycobacterium tuberculosis* is known for long-term survival within the host, and develops multidrug resistance. Before and during infection, the pathogen encounters various harsh environmental conditions. To cope up with such nutrient-limiting conditions, it is crucial to uptake essential nutrients such as ions, sugars, amino acids, peptides, and metals, necessary for numerous vital biological activities. Among the various types of transporters, ATP-binding cassette (ABC) importers are essentially unique to bacteria, accessible as drug targets without penetrating the cytoplasmic membrane, and offer an ATP-dependent gateway into the cell by mimicking substrates of the importer and designing inhibitors against substrate-binding proteins, ABC importers endeavour for the development of successful drug candidates and antibiotics. Alternatively, the production of antibodies against substrate-binding proteins could lead to vaccine development. In this review, we will emphasize the role of *M. tuberculosis* ABC importers for survival and virulence within the host. Furthermore, we will elucidate their unique characteristics to discover emerging therapies to combat tuberculosis.

## Introduction

*Mycobacterium tuberculosis* (Mtb) is one of the world’s most persistent and deadly pathogens. It causes tuberculosis (TB) that remains a major health concern owing to high-mortality rates worldwide. An annual death rate of more than 1.3 million people globally, has been reported due to this disease [1]. The treatment for TB is available, but it takes a period of more than six to nine months. Along with the long-term survival and combination of fixed anti-tubercular drugs, the untimely withdrawal of therapy is the main cause for the emergence of multi, extensively, and even totally drug-resistant strains [2,3]. Currently, only one vaccine i.e. Bacillus Calmette-Guérin (BCG) is available, but its effectiveness varies from very good to no protection in different populations [4]. Therefore, it necessitates determining other targets that could help the discovery of alternate novel drug and vaccine to control and eradicate TB.

Infectious disease and pathogenesis can be considered as the probable ramification of certain pathogenic microorganisms which adopt themselves to cope up with different environmental niches, especially host cells and tissues. Among them, Mtb can persist within the host for a longer period. To resist bacterial colonization at the infection site, host cells adopt various strategies such as antibacterial agents, nutrients limitation, and different stresses [5,6]. In turn, Mtb responds to these multiple stresses by transcriptional reprogramming, for instance, upregulation of oxidative and acid-responsive genes, and metabolic pathway-related genes [7–10]. The upregulation of different nutrient uptake responsive genes at different stages of infection indicates that bacteria utilize different nutrient sources from early to persistent phase. Thus, to make available a variety of substances, necessary for survival, proliferation, and persistence, Mtb acquires these crucial substrates from microenvironments it encounters during infection within the host. This leads to rapid adaptions to the changing host microenvironments, and establishes colonization in the host. However, there is no sufficient information available on Mtb intracellular nutrition, and its link with virulence. Especially, there is a lack of adequate data about the source and uptake mechanisms of essential nutrients like metals, amino acids, sugars, ions, and peptides, the vital virulence determinants to mediate disease. The comprehensive knowledge of host-derived nutrients and mechanisms of uptake and utilization for survival and virulence may help in identifying the key mechanisms for nutritional immunity and metabolic vulnerabilities, nutritional checkpoints in disease progression, reveal metabolic signalling, and most significantly, to uncover the microenvironment at different stages of infection important to develop new chemotherapeutic strategies.

Transporters are chief regulators for the uptake of nutrients, and under different conditions, require around 10–60% of ATP that “clearly shows its essential role in bacterial survival and persistence”. In line with other bacteria, Mtb also utilizes specific transporters, including ATP-binding cassette (ABC) transporters to import (importers) and expel substrates (exporters). It has been determined that 2.5% of the Mtb genome encodes for ABC transporters. In the year 2000, a total of 37 (26 complete and 11 incomplete) ABC transporters with 16 importers and 21 exporters, were reported [11]. Further, in the year 2012 also, a total of 27 complete ABC transporters with 14 importers and 13 exporters, were reported [12]. Exporters have diverse roles such as extrusion of building blocks and the export of various substrates such as lipids, proteins, and antibiotics. It also contributes to the onset of drug resistance through the pumping out the antimicrobial agent. Unlike the ubiquitous ABC exporters, ABC importers mostly in bacteria, are crucial to mediating the uptake of substrates across the cell membrane. ABC importers’ expression and import activity are highly controlled to meet the requirement of nutrients and neutralize toxicity. It also plays a very significant role in the maintenance of cell integrity, differentiation and communication, homeostasis in stress conditions, and also pathogenicity. Several studies on Mtb ABC importer systems depicted their association with physiology and pathogenicity [13–17]. Further, it has also been found that the loss of some ABC importers functions reduced the survival of Mtb, implying that the bacterium cannot grow in the host environment without specific nutrients. Besides this, several drugs against Mtb have failed because of their distinct cell wall composition and organization relative to other similar pathogens, which makes it less permeable. To resolve this major problem, one of the important characteristics of ABC importers, is that it acts as an ATP-dependent gateway into the cell, and therefore, could be utilized for efficient delivery of antimicrobials.

Thus, ABC importers can offer a new paradigm to identify targets and develop effective drugs and antibiotics by mimicking transporter substrates and designing inhibitors that specifically target substrate-binding proteins. Besides this, it could help in the development of successful vaccines through the production of antibodies against substrate-binding proteins. Therefore, here we provided a systematic inventory of Mtb ABC importer systems and their role at the host–pathogen interface. Further, we also discussed their possible roles as targets for the delivery of antimicrobials and the development of drugs and vaccines. It is anticipated that this review will encourage further research for an in-depth understanding of the transport systems viz. their structure and mechanisms which are essential for the commencement of new therapeutic agents.

## Architecture and mechanism of bacterial ABC transporter

**Figure 1.**
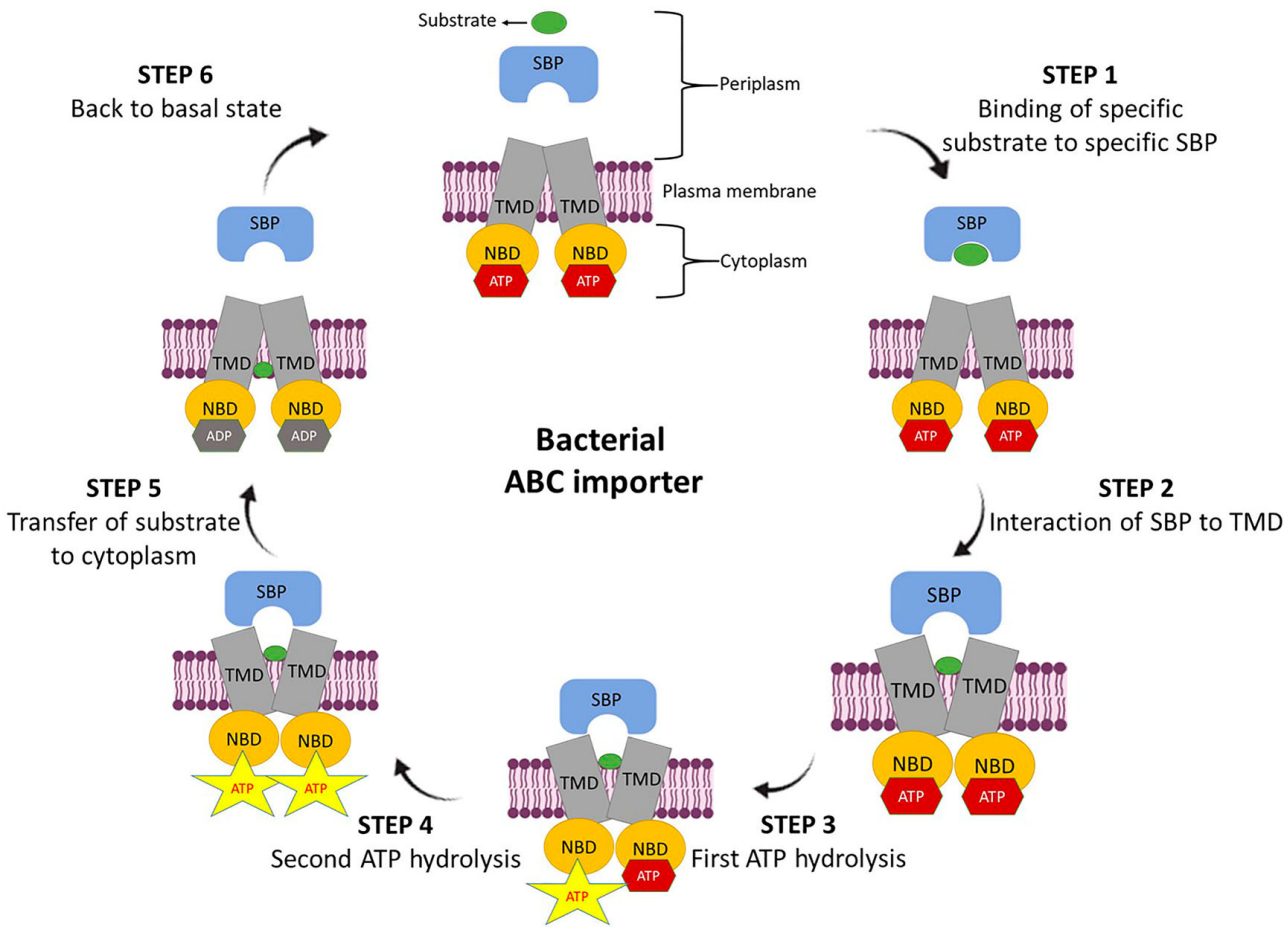
Schematic representation of ABC import system. Step 1. In periplasm, substrate-binding protein (SBP) binds to its respective substrate to form SBP-substrate complex. Step 2. Interaction of SBP-substrate complex to transmembrane domains (TMDs) leads to outward-facing confirmation and release of the substrate into the TMDs binding cavity. Step 3. First, ATP hydrolysis at consensus site of one nucleotide-binding domain (NBD). Step 4. Second ATP hydrolysis at second consensus site of second NBD. Step 5. Conformational changes in TMDs leads to inward-facing conformation that allows translocation of the substrate into the cytosol. Step 6. Importer back to its basal state by nucleotide exchange.

ABC transporters are ubiquitous to all the kingdoms of life, and are considered as one of the largest classes of transporter superfamily. On the basis of their transport directions i.e. either inside or outside of the cell, they are characterized as importer and exporter, respectively. However, both of them have almost the same typical structural design with the minimum four domains, two each of hydrophobic transmembrane domains (TMDs, embedded in membrane bilayer) and nucleotide-binding domains (NBDs, located in the cytoplasm). Additionally, importers also have a high-affinity extra-periplasmic (Gram-negative bacteria) or lipid-anchored external (Gram-positive bacteria) substrate-binding protein (SBP). The general mechanisms of substrate translocation across membranes are also nearly the same for both of them. The schematic representation of the ABC import system is shown in Figure 1. In brief, in periplasm, SBP bind to the specific substrate, and deliver it to the respective importer system. Thereafter, hydrolysis of two ATP molecules occurs at two consensus sites which lead to conformational changes in TMDs allowing the translocation of substrate into the cytosol.

The structural and functional characteristics of the ABC transporters have been extensively reviewed [18–20]. Briefly, the NBDs of all ABC transporters possess highly conserved stretches of amino acids and several characteristic motifs (the Walker A (GXXGXGK(S/T)), Walker B (ϕϕϕϕD, ϕ is the hydrophobic residue) and signature motifs (LSGGQ, the hallmark of the ABC family) along with the Q, H, and D-loops, irrespective of their origin (prokaryotic or eukaryotic), transport directions (import or export), substrate specificity, and physiological role. In the folded NBDs, these conserved residues/motifs are arranged to create two ATP-binding sites between the Walker A and the signature motif of the other. The function of these amino acid residues and motifs, like ATP binding and hydrolysis and to power transmission, is also highly conserved. For example, glutamate in the Walker B motif and aspartate, histidine, and glutamine in the Walker B motif, H-loop, and Q-loop, have similar functions (catalytic residue for ATP hydrolysis) not only in exporters and importers but also in prokaryotic and eukaryotic ABC transporters. In contrast to NBDs, TMDs are less conserved, while the presence of a highly conserved motif (EAA motif) is notified in the cytoplasmic loop of NBDs. The EAA motif interacts with the Q-loop of the NBD and thereby, is the crucial component in coupling NBDs to TMDs.

While the general architecture and mechanism is conserved among ABC transporters, several classes of ABC transporters have emerged as more systems are characterized (for comprehensive reviews see [21,22]). Briefly, Type I importers (for maltose transporter, MalFGK2; methionine, MetNI and molybdate, ModBC) permit medium-affinity import of nutrients, ions, sugars, amino acids, small peptides, and oligosaccharides [23–25]. Further, the substrate-binding pocket in type I importers is connected by flexible loops (permits free movement of the lobes relatively) while re-orientation of TMDs from inward- to outward-facing conformation involves conformational changes in rigid body on a large-scale. Whereas, type II importers (such as vitamin B12 transporter (BtuCD), heme transporter (HmuUV) and molybdate transporter MolBC (HI1470/1)) permit high-affinity uptake of metal chelates such as heme and other iron-containing complexes, and also cobalamin [26–28]. Apparently, the substrate-binding pockets in type II importers are connected by rigid α-helix structures (block the relative movement of the lobes) and there occurs alternating access (and occlusion) via the orchestrated local residues movements that function as gates. Furthermore, intergroup speciation has also been seen between type I and II transporters. For example, three Type II transporters (MolBC, BtuCD, and HmuUV) appear to have highest, intermediate and lowest level of uncoupled ATPase activity, respectively [26–28]. From these studies, it is anticipated that there exist distinct defined clusters that facilitate the continuum of mechanistic speciation and adaptations in the transporters.

## ABC importer systems of *M. tuberculosis*

On the basis of structural similarities between ABC transporters in all living organisms, a total of 16 Mtb ABC importers were reported [11]. Further, by the year 2012, a total of 14 complete Mtb importers are reported [12]. Here, in this review, we cover all the importers in detail and the major findings are summarized in Table 1. The below sections comprise detailed information on Mtb ABC importer systems like amino acid, sugar, metal, and anion, and their role at the host–pathogen interface.

**Table 1.** ATP-binding cassette (ABC) import systems of *M. tuberculosis* with the major findings.

ABC importers	Components of ABC importer	Major findings
*Amino acid importers*		
Oligopeptide importer (OppABCD/Rv1283c-Rv1280c)	SBP: OppA (Rv1280c) TMD: OppB (Rv1283c) and OppC (Rv1282c) NBD: OppD (Rv1281c) (two copies)	*oppA* shows broad substrate specificities [17] *oppD* mutant shows reduced survival in macrophages [30] *oppD* mutant shows decreased apoptosis-inducing ability and cytokines production [17]
Dipeptide importer (DppABCD/Rv3666c-Rv3663c)	SBP: DppA (Rv3666c) TMD: DppB (Rv3665c) and DppC (Rv3664c) NBD: DppD (Rv3663c) (two copies)	*dppD* mutant shows decreased survival in mice [14] *dppC* mutant attenuated for virulence in mice [31] *dppABCD* knockout strain shows reduced survival in mice [29]
Glycine betaine importer (ProXVWZ/Rv3759c-Rv3756c)	SBP: ProX (Rv3759c) TMD: ProW (Rv3757c), and ProZ (Rv3756c) NBD: ProV (Rv3758c) (two copies)	*proXVWZ* mutant shows decreased survival in macrophages [15] Import polyphenols instead of glycine betaine [32]
Glutamine importer (Rv2563, GlnQ/Rv2564, Rv0072, Rv0073, and GlnH/Rv0411c)	SBP: GlnH (Rv0411c) TMD: Rv2563 and Rv0072 NBD: GlnQ (Rv2564) and Rv0073	Experimentally not characterized.
*Sugar importers*		
Glycerophosphocholine importer (UgpABCE /Rv2832c-Rv2835c)	SBP: UgpB (Rv2833c) TMD: UgpA (Rv2835c) and UgpE (Rv2834c) NBD: UgpC (Rv2832c) (two copies)	UgpB is a substrate of the twin-arginine translocation (Tat) pathway [39] Import glycerophosphocholine instead of sn-glycerol-3-phosphate and maltose [39]
Trehalose importer (LpqY-SugABC/Rv1235-Rv1238)	SBP: LpqY (Rv1235) TMD: SugA (Rv1236) and SugB (Rv1237) NBD: SugC (Rv1238) (two copies)	*LpqY-SugABC* mutant shows reduced virulence in mice [43] Uptake and recycle disaccharide trehalose [43] Involved in biofilm formation [43]
Amino-sugar importer (UspABC/Rv2316-Rv2318)	SBP: UspC (Rv2318) TMD: UspA (Rv2316) and UspB (Rv2317) NBD: Remains to be identify	Essential for *in vitro* growth [50] UspC have a substrate preference for sugars containing an amino group at the C2 or C3 position [36]
Rv2038c-Rv2041c	SBP: Rv2041c TMD: Rv2039c and Rv2040c NBD: Rv2038c (two copies)	Rv2041c treatment with macrophages and lymphocytes shows elevated expression of pro-inflammatory cytokines and raised up secretion of IFN-γ and TNF-α, respectively [52] Rv2041c gives positive antibody responses and high level of sensitivity with Mtb infected mice [53]
*Metal importers*		
Iron importers (IrtA/Rv1348, IrtB/Rv1349, FecB2/Rv0265c, and FecB/Rv3044)	SBP: FecB2(Rv0265c) TMD: Remains to be identify NBD: Remains to be identify	*fecB2* mutant shows reduced growth in medium containing heme as a sole iron source [60]
	SBP: FecB (Rv3044) TMD: Remains to be identify NBD: Remains to be identify	*fecB* shows maximum expression in absence of iron from media [58]
	SBP: Remains to be identify TMD: IrtA (Rv1348)^a^ and IrtB (Rv1349)^a^ NBD: IrtA (Rv1348)^a^ and IrtB (Rv1349)^a^	Involved in maintaining balance of siderophores [16,57]
*Anion importers*		
Sulphate importer (SubI-CysT-W-A/Rv2397c-Rv2400c)	SBP: SubI (Rv2400c) TMD: CysT (Rv2399c) and CysW (Rv2398c) NBD: CysA (Rv2397c) (two copies)	Induced expression of *cysT* and *cysA1*, *cysT*, *cysW*, and *subI* genes in hydrogen peroxide and nutrient starvation conditions, respectively [64–66]
Molybdate importer (ModA/Rv1857)	SBP: ModA (Rv1857) TMD: ModB (Rv1858) (two copies) NBD:ModC (Rv1859) (two copies)	*modA* mutant shows decreased survival in mice [69]
Phosphate importers (PstS3-C2-A1/Rv0928-Rv0930, PstS2-PknD/Rv0932c-Rv0931c, and pstB-S1-C1-A2/Rv0933-Rv0936)	SBP: PstS3 (Rv0928) TMD: PstC2 (Rv0929) and PstA1 (Rv0930) NBD: Remains to be identify	PstS3 import system shows role in virulence and survival within macrophages [44]
	SBP: PstS2 (Rv0932c) TMD: Remains to be identify NBD: Remains to be identify	Knockout of *pstS1* and *pstS2* shows decreased survival and attenuated for virulence in mice [74] PstS1, an immunodominant antigen, activate TNF-α and IL-6 in human primary monocytes [75,78]
	SBP: PstS1 (Rv0934) TMD: PstC1 (Rv0935) and PstA2 (Rv0936) NBD: PstB (Rv0933) (two copies)	

Note: SBP: Substrate-binding protein; TMD: Transmembrane domain; NBD: Nucleotide-binding domain.

^a^contains both TMD and NBD as one polypeptide.

## Amino acid importers

Amino acids as the carbon and nitrogen sources, are the essential building block for the cell. During infection and/or the limitation of nutrient, pathogenic bacteria, for example, Mtb requires amino acids from the host through active transport. Several ABC transporters facilitate uptake of amino acids and meet the metabolic requirements during growth, persistence, and virulence. Thereby, these have a central role in metabolic interactions between host and the pathogen. In Mtb, some amino acid ABC transporters have been functionally characterized (ProXVWZ, OppABCD, and DppABCD). The one predicted as glutamine ABC transporter remains to be explored yet.

### Peptide importer (OppABCD/Rv1283c-Rv1280c and DppABCD/Rv3666c-Rv3663c)

Peptide importers facilitate uptake of specific peptide sequences involved in sporulation, aerial hyphae formation, competence, and virulence. This establishes their role in bacterial adaptations to diverse conditions. In Mtb, two ABC peptide importers, OppABCD (oligopeptide importer, Rv1283c-Rv1280c), and DppABCD (dipeptide importer, Rv3666c-Rv3663c), have been predicted [11]. However, functional characterization of Rv1280c in H37Rv strain showed broad substrate specificities such as tripeptide glutathione and nonapeptide bradykinin [17]. Further, it is suggested that Rv3666c-Rv3663c transport system was involved in the import of oligopeptides and lipopeptides [29]. Therefore, more study is warranted to identify the substrate specificity of such transporters.

Several studies described the role of these transporters in the survival of mycobacteria. The disruption of Rv1281c in *Mycobacterium bovis* BCG showed decreased resistance to toxic peptides such as glutathione and S-nitrosoglutathione, and reduced survival in cultured inactivated murine macrophages [30]. Dasgupta et al. [17] also reported Mtb Rv1283c-Rv1280c transport system to have a role in the regulation of glutathione and methyl glyoxal levels. They also showed that inactivation of Rv1281c decreased apoptosis-inducing ability and production of cytokines IL-1b, IL-6, and TNF-α.

The deletion of Rv3663c in H37Rv strain showed decreased survival at the early stage of mice infection [14]. Likewise, the disruption of Rv3664c in BCG strain resulted in most attenuating mutations [31]. Flores-Valdez et al. reported that knockout strain of Rv3666c-Rv3662c transport system had reduced survival in mice lungs and spleen during the active stage of infection but did not show differential virulence phenotype [29]. They suggested that this transport system was involved in the regulation of intracellular signalling pathways that support Mtb survival within the host. This system regulates the expressions of many genes (*fasI*, *desA3*, *icl*, *fadE13*, and *PE13*) that encode for mycolic acids, PDIMs as well as PE-family proteins and thereby, provide the signals necessary for remodelling the cell wall components. Therefore, further studies on the peptide importers may help in finding such signalling molecules that regulate the active and dormancy stage of mycobacteria.

### Glycine betaine importer (ProXVWZ/Rv3759c-Rv3756c)

Osmoregulation plays an important role in bacteria and helps them cope up with the diverse harsh conditions and establish host infection. The different *in vivo* environments encountered by Mtb during infection widely vary in terms of available water (i.e. water activity). Price et al. reported that for maintaining osmotic balance, Mtb imports glycine betaine from host macrophages through the ProXVWZ transporter system [15]. They also found upregulation in *proXVWZ* expression in response to phagocytosis, and suggested that the system supports Mtb survival at raised osmolarity. They also reported that the deletion of *proXVWZ* operon led to the loss of betaine accumulation under osmotic stress, and Mtb showed decreased growth within human macrophages. On the contrary, Zhao et al. crystallized ProX and found no interaction between ProX and betaine, choline or carnitine [32]. Further, they demonstrated the implication of ProX in the import of polyphenols such as phloretin, monoacetylphloroglucinol, and 2,4-dihydroxyacetophloroglucinol or analogous compounds. This study indicates that Mtb ProXVWZ transport system involved in the import of polyphenols instead of glycine betaine can be used as the carbon source. In addition, they also somehow, regulate the accumulation of betaine under osmotic stress and have an important role in survival within human macrophages. Thus, further studies are imperative to identify the substrate specificity, and explore the function(s) of this transporter system.

### Glutamine importer (Rv2563, GlnQ/Rv2564, Rv0072, Rv0073, and GlnH/Rv0411c)

Glutamine is required for many essential biological processes such as biosynthesis of nitrogen-containing compounds and synthesis of protein. Thus, regulations of glutamine uptake and catabolism have a role in bacterial adaptability. It is expected that studies on glutamine regulators (specifically, the glutamine ABC importer) and their metabolic pathways would be advantageous for exploring bacterial adaptability and virulence. In the year 2000, glutamine ABC importer was predicted in Mtb, however, its experimental identification and characterization need to be explored [11].

## Sugar importers

Carbohydrates and lipids have a pivotal role as the building block, and in bacterial survival and adaptability. It was reported that Mtb survival in mice depended on the glyoxylate cycle, and it renewed attentiveness towards carbon metabolism [33,34]. The assessment of carbohydrate ABC importer system in Mtb revealed that it to have four carbohydrate ABC import systems (UgpABCE, ABCSug, ABCUsp, and Rv2038c-Rv2041c systems) [35,36]. These systems have very low (25%) similarity with the known transporters in different bacterial species and that leads to unpredictability for the substrates used by them. Therefore, identification and characterization of each carbohydrate ABC import system are the requisite.

### Glycerophosphocholine (GPC) importer (UgpABCE /Rv2832c-Rv2835c)

Mtb UgpABCE importer system has similarity with the maltose system of *Escherichia coli*. As a consequence, this system is grouped with CUT1 (carbohydrate uptake transporter-1) family and predicted as sn-glycerol-3-phosphate (G3P) binding proteins [37,38]. Contrary to these hypotheses, no detectable binding activity was observed for Mtb UgpB with G3P and maltose [39]. The crystal structure comparison of UgpB depicted that the site (Trp 169) essential for G3P binding in *E. coli*, is replaced in Mtb by Leu 205. Further, the investigator suggested that Leu205 was the determining factor for glycerophosphocholine (GPC) binding since mutation of Leu 205 terminated GPC binding [39]. This study specifies the role of Mtb UgpB in the import of GPC which can act as the carbon and phosphate source. Besides this, Mtb UgpB is also the substrate of twin-arginine translocation (Tat) pathway, essential for virulence and survival in several pathogens [40,41]. However, further studies are imperative to establish its role in Mtb virulence and survival.

### Trehalose importer (LpqY-SugABC/Rv1235-Rv1238)

It was predicted that Mtb LpqY-SugABC system was involved in the import of maltose or maltodextrins [11,42]. But, studies indicate that this system has less similarity (25%) with MalE (maltose transporter) of *E. coli* and *S. coelicolor*, and Mtb is not able to grow in media with maltose as the sole carbon source. Nevertheless, it has been revealed that the Mtb LpqY-SugABC importer was very specific for the uptake and recycling of the disaccharide trehalose, a sugar absent in mammals, and therefore, it was incapable of nutrient acquisition from the host [43]. It was also reported that disruption of this function leads to reduced virulence, and this is in agreement with previous findings that this system was essential for the growth of Mtb in macrophages as well as in mice [14,44].

Additionally, the release of free mycolic acid through the metabolism of trehalose establishes its role in the formation of mycobacterial biofilm. Hence, several inhibitors have been developed targeting trehalose metabolism. The first inhibitor 6-azido-6-deoxy-α,α׳-trehalose (6-TreAz, 8) tested on *Mycobacterium aurum in vitro*, showed reduced growth [45]. Also, in Mtb, the reduction in growth was observed by chemically synthesized trehalose analogues [46]. Recently, to make simple analyses of different structures, the “chemoenzymatic method” has been developed [47]. By utilizing this method, investigators reported some novel trehalose-based inhibitors that retarded *Mycobacterium smegmatis* growth and biofilm formation. It was suggested that antimicrobial and anti-biofilm activities of these inhibitors rely on their uptake by trehalose importer LpqY-SugABC. But in case of Mtb, no reduction in biofilm was seen by the deletion of trehalosedimycolate hydrolase that mediates the release of free mycolic acid [48]. Therefore, at present, it is unclear that this strategy could be applied or not on Mtb. However, apart from trehalosedimycolate, other byproducts such as trehalose monomycolate and arabinogalactan mycolate are associated with trehalose metabolic pathways, and thereby, with the cell wall components. Further studies targeting such compounds are warranted to explore their role in the inhibition of Mtb biofilm.

### Amino-sugar importer (UspABC/Rv2316-Rv2318)

This importer system consists of *uspA* and *uspB* as a TMD and *uspC* as an SBP. However, NBD is still unknown, and it is believed that it shares NBD of another ABC transporter [11]. In different mycobacterial species, *uspABC* is conserved [49]. Further, it has been shown to be essential for *in vitro* growth [50]. These characteristics suggest *uspABC* to be considered among the core genes required for the intracellular survival of this pathogen within the host. Recently, Fullam et al. reported that Mtb UspC has two subdomains, which makes a highly acidic carbohydrate-substrate-binding cleft [36]. This study also showed Mtb UspC to have preference for sugars with an amino group at the C2 or C3 position, and act as an amino-sugar transporter. It is anticipated that during the infection phase especially inside the phagosomes where carbohydrates are limited, this transport system could help optimize the use of carbohydrates. This indicates that Mtb UspABC transport system might be essential for intracellular survival, and which, can further be explored.

### Rv2038c-Rv2041c

This transport system (Rv2038c-Rv2041c) comprises an SBP (sugar-binding lipoprotein) predicted for the import of carbohydrate, but its exact substrate specificity is yet to be established. However, the upregulation of Mtb Rv2041c in acidic and hypoxic conditions that Mtb face *in vivo* (specifically in phagosome) suggests its role in intracellular adaptation within the host [51,52]. It was reported that Rv2041c treatment with macrophages resulted in elevated expression of pro-inflammatory cytokines (TNF-α, IL-6, and IL-12p40) [51,52]. Further, the treatment of this ABC importer component with lymphocytes from latent and active TB mice raised up the secretion of IFN-γ and TNF-α. In addition, it was reported that Rv2041c gives positive antibody response with Mtb infected mice as well as active TB patients [53]. It was also established that this protein provides the highest sensitivity similar to previously used serological antigens (CFP-10, ESAT-6, HSP-X, Ag85 complex, and PstS1) against active Mtb infection. In view of the literature available, it can be anticipated that Rv2041c strongly induces a cellular immune response, and could be used as T-cell antigen for the serodiagnosis of Mtb infection, although further investigations have to be carried out to understand these aspects.

## Metal importers

Metals have a very crucial effect on the intracellular growth of bacteria, and loss in metal uptake by ABC importers had lethal effects on the virulence of the pathogenic bacterium. There are reports on the presence of at least four iron ABC importers encoded by the Mtb genome.

### Iron importer (IrtA/Rv1348, IrtB/Rv1349, FecB2/Rv0265c, and FecB/Rv3044)

Many pathogenic bacteria, including mycobacteria, can procure nearly all nutrients excluding iron. Among various approaches adopted by Mtb for iron uptake, one is the release of siderophores i.e. carboxymycobactin and lipophilic mycobactin [54,55]. It was found that under iron-limiting conditions, levels of cellular siderophores and corresponding transport protein are increased which illustrates their role in iron uptake [54,56]. Some studies reported the involvement of two IdeR (iron-dependent regulator) controlled ABC importers, IrtA (act as a carboxymycobactin exporter) and IrtB (act as a two-component importer of ferri-carboxymycobactin) in iron uptake [16,57]. They also suggested that three mycobacterial proteins i.e. IrtA, IrtB, and Rv2895c are synergistically involved in maintaining the balance of siderophores, and thereby in iron acquisition and survival of Mtb. In addition, FecB and FecB2, putative iron (III) dicitrate binding proteins, are iron importers. In a study with *M. avium*, *fecB* expression was poorly correlated with alterations in the iron concentration of media, however, in the absence of iron, the protein showed maximum expression [58]. Further, in the same study, there was no *fecB* induction during initial infection in macrophages. This suggests that iron concentration in mycobacteria was not low enough to stimulate the *fecB* expression. However, further studies in this aspect are required to prove *fecB* interaction with mycobactin and carboxymycobactin and to explore its involvement in siderophore mediated or citrate dependent iron transport pathway.

It is assessed that about 70% of iron content in the human body is associated with heme, preferably in the form of haemoglobin, which cannot be solubilized by siderophores. Thus, another approach that many pathogenic bacteria, including Mtb, may adopt for iron acquisition is, from heme. The crystal structure of Mtb FecB2 (PDB id: 4PM4) depicted a high level of similarity with HmuT of *Yersinia pestis* [59]*.* HmuT is a periplasmic protein of *Y. pestis* ABC importer system, and involved in the uptake of heme. Further, in a recent study, it was evaluated that the growth of Mtb *fecB2* mutant relative to the wild type, was reduced in medium containing heme as the sole iron source [60]. These findings suggest its role in heme utilization for iron acquisition. Overall, these findings indicate the involvement of different ABC importers in iron uptake, and their contribution to adaptation and survival. However, future investigations are warranted to completely unravel iron acquisition mechanisms.

## Anion importers

Anions like sulphur, molybdate, and phosphate are crucial for survival, and play an important role in virulence of many pathogenic bacteria. They have a main role in the biosynthesis of molecules, and are also involved in several biological activities. One sulphate, one molybdate, and three phosphate ABC importers are reported in Mtb [11].

### Sulfate importer (subI-cysT-W-A/Rv2397c-Rv2400c)

Sulphur is requisite for many biological processes such as translation initiation, protein formation, cysteine and methionine biosynthesis, and redox maintenance. These activities are essential for survival, and also for virulence in several pathogens together with Mtb. Besides this, microbial sulphur metabolic pathways are not present in humans. These properties make it a unique target for combating with Mtb infection. The assimilation of sulphate starts with active uptake of inorganic sulphate by the corresponding ABC importer [61]. In a study with *M. bovis* BCG, it was found that *subI* and *cysA* mutants had reduced growth, and this could be overcome by the addition of methionine but not cysteine, and that is in contrary with *cysH* mutant of *M. smegmatis* and Mtb [61]. Further, in the presence of 0.3 mM cysteine, wild-type *M. bovis* BCG grew slowly while in presence of still higher dose (0.5 mM), no growth was observed. These results are in contrary with the observations in wild-type strains of *M. smegmatis* or Mtb grown in 1–2 mM cysteine where no toxicity has been found [62,63]. These observations indicate that mutation in *M. bovis cysA* gene results in complete inhibition of sulphate uptake, and renders mutant auxotrophic to methionine characteristics. Above studies provide evidence for the presence of alternate sulphate transporters that get activated during infection, and the existence of other biochemical pathways that feed into the cysteine biosynthetic pathway.

Genes that coordinate sulphate transport and the first few steps of sulphate assimilation are overexpressed in Mtb subjected to a variety of stress conditions. For example, *cysT* was induced by a stimulus with hydrogen peroxide. Under nutrient starvation, *cysA1*, *cysT*, *cysW*, *subI* genes encode for sulphate transporter complex [64–67]. Some frontline antibiotics used to treat TB also induce genes of the sulphate assimilation pathway. For example, menadione (a vitamin K precursor that promotes the production of reactive oxygen species) induces *cysA1*, *cysT*, *cysW*, *subI* genes [67,68]. These data suggest that genes involved in sulphite assimilation have an important role in the survival of this pathogen, and also the response to antibiotics used in current treatment regimes, although, further investigations are required to establish this fact.

### Molybdate importer (ModA/Rv1857)

Molybdate acts as a cofactor present in the active site of various enzymes, and plays a role in carbon and nitrogen cycling. It is reported that a mutant of molybdate ABC importer gene “*modA*” had decreased survival in mice lungs [69]. This observation depicted that uptake of molybdenum was required for the survival of this pathogen. Further, several enzymes form the molybdenum cofactor (MoCo), and molybdenum is harmonized with the dithiolene group of molybdopterin [70]. Thus, lack of molybdenum import may affect the biosynthesis of MoCo, and consequently, lead to a functional deficiency in the majority of the enzymes dependent on molybdenum for the survival of the pathogen. Hence, more studies are warranted to establish the role of these enzymes in Mtb survival and virulence.

### Phosphate importer (pstS3-C2-A1/Rv0928-Rv0930, pstS2-pknD/Rv0932c-Rv0931c, and pstB-S1-C1-A2/Rv0933-Rv0936)

Phosphorus is needed by all cells for the synthesis of important biological molecules such as phospholipids and nucleic acids, and also for energy metabolism. Bacteria mainly acquire phosphorus from inorganic phosphate, and have an inducible, high-affinity ABC transport system Pst (phosphate specific transport). Mtb contains three Pst system: PstS1, PstS2, and PstS3. Mtb PstS1 is mainly located in the cell wall and on the outer surface [71]. Overexpression of PstS1 has also been reported under phosphate limiting conditions [72,73]. Further, in a study by Peirs et al., they reported that knockout of *pstS1* and *pstS2* led to a substantial reduction in growth rate in mouse peritoneal macrophages [74]. This study also reported that these mutants were attenuated in an *in vivo* infection model. They also performed experiments with media having low concentrations of phosphate, and established the role of these two Pst systems (PstS1 and PstS2) in phosphate import which is important for intracellular survival. Similarly, in another study, Rengarajan et al. found that PstS3 import system plays a role in virulence, through in the import of phosphate and survival within macrophages [44]. Nevertheless, additional work on this aspect is required to establish its role in Mtb virulence and survival.

Moreover, PstS1 has been previously described as glycoproteins, and then, as glycolipoprotein [75,76]. It has been reported that in TB patients, PstS1 acts like an immunodominant antigen. It also has a motif that interacts with both Toll-like receptor2 (TLR2) and TLR4, and activates ERK1/2 and p38 MAPK [77,78]. This further leads to the activation of TNF-α and IL-6 in human primary monocytes. From these reports, it can be anticipated that this glycolipoprotein acts as an immunodominant antigen, and possibly during infection, plays a role in early inflammatory responses, however, further investigations are required to ascertain this fact.

## Discussion

**Figure 2.**
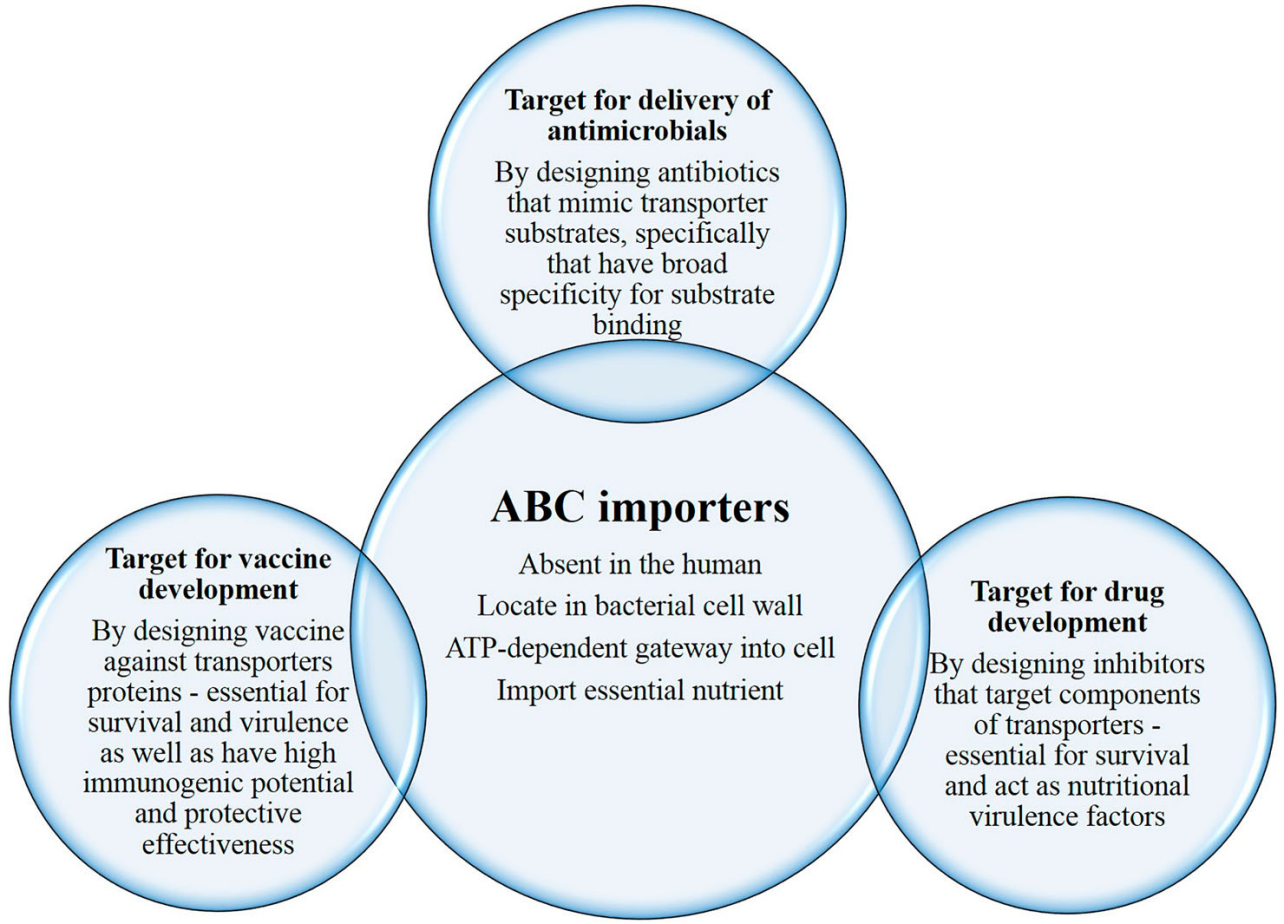
ABC importer features along with unique characteristics that makes them ideal target for the delivery of drugs, and also for the development of novel drug(s) and vaccine(s).

The severity of TB caused by Mtb, is life-threating for humans, and a further increase in antibiotic resistance makes this even more dangerous. As highlighted in this review, mycobacteria persist inside the host with diverse residency: phagosome, phagolysosome or cytosol, and from early to late *in vivo* infection where it comes across with various physiological conditions such as acidic stress, bile stress, nitrosative stress, and nutritional deprivation. Such altering locations and microenvironments necessitate uptake of specific substrates to combat these situations. Hence, ABC importers that acquire essential nutrients, metals, and ions through active transport, could have an important role for this pathogen in survival and persistence within the host. The detailed knowledge of their structure and mechanism of substrate translocation, along with the suitability of the overall survival mechanism could open a new avenue to combat this deadly disease. Therefore, the purpose of this review is to provide the summary of current information for Mtb ABC importers. Figure 2 shows features of ABC importer along with unique characteristics that make them the ideal target for the delivery of drugs, and also for the development of novel drug(s) and vaccine(s).

## ABC importers – target for delivery of antimicrobials

The role of ABC importers is well established in the uptake of the essential nutrients. Nevertheless, it can also play a role as an ATP-dependent gateway into the cell, and these characteristics open a new possibility to utilize them as a system for delivery of antimicrobials (antibiotics that mimic transporter substrates) into bacterial cells. As presented in this review, Mtb importers such as ProX and DppA system have notable ability to bind with a wide range of substrates [17,32]. The broad specificity of these transporters makes them ideal to exploit for the delivery of novel antimicrobials. Earlier reports provided proof of concept for this approach, for example naturally synthesized antibiotics such as microcin C (blocks the synthesis of tRNA^asp^) and pacidamycin (inhibits translocase I, MraY) are imported to the cell by NppA1A2BCD system in *Pseudomonas aeruginosa* and YejABEF system in *E. coli*, respectively [79–83]. In addition, the role of ABC importers has also been demonstrated in the import of synthetic antibiotics such as phaseolotoxin and kasugamycin that inhibit orthine carbamoyltransferase and translation initiation, respectively, are imported via DppABCDF in *E. coli* [84,85]. Similarly, it is found that kasugamycin and translation termination inhibitor blasticidin are imported via OppABCDF [85]. In view of these findings, it is clear that this feature of ABC importer has significant potential, and can be exploited for the delivery of antimicrobials. As a result, in case of Mtb, where the main reason for the failure of many candidate antimicrobials is their incapability to breach the cell wall, the selection of antibiotics that mimic transporter substrates, could greatly increase the chance for finding the successful drug candidates. However, further studies are warranted to exploit ABC importers specifically SBP as the target, for the delivery of antimicrobials.

## ABC importers – target for drug development

Several studies established direct or indirect participation of Mtb ABC importers in survival and virulence of bacterium within the host. Like, in *in vivo* experiments, UgpB and LpqY-SugABC have been shown to be essential for growth and virulence, respectively [13,14]. The deletion of *oppD* resulted in decreased survival in early mice infection [14]. Further, the deletion of *proXVWZ* operon resulted in reduced growth within human macrophages [15]. In addition to nutrients, it also concomitantly helps in the uptake of metals and anions, and these Mtb importers are also involved in survival and virulence of bacterium within the host. For example, an *in vivo* study showed that *modA* mutant had reduced survival in lungs of mice [69]. Further, *irtAB* mutant has been also shown to have affected growth in human macrophages and lungs of mice [16]. Collectively, these studies provide evidence that ABC importers are nutritional virulence factors, and essential for mycobacterial survival and virulence. In addition, they are absent in the host and located in the bacterial cell wall which makes, them the ideal target for drug development. It is therefore, hypothesized, that the components of ABC importer could be the potential drug targets that could restrict nutrient uptake, and in turn, disrupting the virulence pathways of pathogens. An existing example is ZnuABC of *Salmonella typhimurium* wherein two zinc-binding compounds, RDS50 and RDS51, inhibited growth and decreased Caco-2 cells invasion [86]. Additionally, therapeutics need to be directed in targeting the TMD pathway of translocation and ATP hydrolysis of NBD to further avert transport of nutrients and bacterial pathogenesis. The existing literature reveals that function loss in certain ABC transporters leads to loss of virulence, thereby increasing the scope of therapeutics targeted towards ABC importer. However, future studies in this direction would expand the possible avenues in designing drugs against infections.

## ABC importers – target for vaccine development

As discussed above, some of the ABC importers play role in bacterial virulence and survival thus specifying the components of these importers as ideal targets for mutational development of active attenuated antibacterial vaccines. In addition, ABC importers also have immunogenic potential that opens the possibility to utilize them as candidate subunits for vaccination by production of antibodies against the SBPs. For example, Mtb PstS is highly immunogenic in intramuscular injection to mice as the DNA vaccine, and also has protective effectiveness against intravenous administration with Mtb [87]. The inactivation of Rv1281c leads to reduced apoptosis and compromised inducing ability of cytokines (IL-1b, IL-6, and TNF-α) [17]. Immune response to Mtb Rv2041c protein treatments with macrophages resulted in elevated expression of TNF-α, IL-6, and IL-12p40 [52]. Further, treatment of Rv2041c protein with lymphocytes from latent and active TB mice raised up the secretion of IFN-γ and TNF-α. Moreover, they indicated positive antibody responses to Rv2041c only in active TB patients and Mtb infected mice [53]. Proof of concept for this approach has also been seen in other bacterial species, as the mutant of *exsA* (component of ABC transporter) in *Brucella abortus* reflected immunity in mice against virulent *B. abortus* challenged with mice immunized with commercial vaccine [88]. Further, co-immunization of *Streptococcus pneumoniae*, PiuA and PiaA, revealed that the deployment of two iron SBPs antigens had additive protection in mice [89]. Furthermore, immunization with the recombinant manganese SBP, MntC, elicited anti-MntC IgG response in terms of protecting mice against *Staphylococcus aureus* [90]. Additionally, the *S. aureus* vaccine successful in clinical trials, harbours multiple antigens, and targets various virulence mechanisms, including MntC [91]. These reports strengthen that some of the ABC importers modulate innate immune response and have a role in apoptosis of host macrophages, the prerequisite for an effective immune response. Therefore, these provide evidence towards the suitability of ABC transporter proteins for the development of antibacterial vaccines, either through live attenuated bacteria or by the designed protein – and DNA-based subunit vaccines. However, further studies in this direction are needed for the rational approach for the assortment of ABC importers as candidate vaccine antigens.

Although the above studies indicated the importance of ABC components as the potential therapeutic targets, there are several challenges associated with their architecture, mechanism, and selectivity for the substrate. Therefore, the main challenge will be to obtain high-resolution structures of SBPs with the ligand as well as of the ABC component from different intermediate steps of the transport cycle.

## Conclusions

It is apparent that transporters especially ABC ones involved in the uptake of essential nutrients in mycobacteria, are not well studied, regardless of their significant role in Mtb physiology and pathogenicity. The reports summarized in this review indicate various important characteristics of Mtb ABC importers like – (1) acquire essential nutrients from the host, and it is depicted that loss of this function by some of the Mtb ABC importers results in decreased virulence, survival, and persistence. This indicates that such a feature can be utilized in the development of novel drug either by targeting SBP or inhibit the interactions between SBP and TMD as well as the interaction between TMD and NBD to restrict nutrient uptake by the pathogens, (2) In addition, ABC importers act as an ATP-dependent gateway for specific substrates, and in mycobacteria, many candidate antimicrobials have been unsuccessful because of their failure to cross the cell wall. In view of this, this feature can be utilized for the delivery of inhibitors against the SBP. Further, as discussed in this review, some Mtb importers also have wide specificity for substrates, which makes them ideal for designing new inhibitors, (3) Furthermore, ABC import systems are located in the membrane and several proteins of this system are exposed to the surface; such characteristics increase the chance of having immunogenic properties. Some of Mtb ABC importers proteins possess the immunogenic potential, and thereby, offer ideal candidates for vaccine development, and (4) ABC importers are absent in mammalian host, and this increases the promise of ABC importer for targeted therapeutics.

Although researches on ABC import systems are still in their infancy, these systems show potential as drug targets to prevent tuberculosis. The combination of high-resolution structures with biochemical and genetic analyses of Mtb ABC importers will improve our knowledge for physiology and pathogenicity of this deadly pathogen and further, it could be used to improve tuberculosis chemotherapy. Further, as ABC import systems are ubiquitously present in bacteria, they offer targets for a wide spectrum of potential novel drugs. It is anticipated that this review will motivate for more investigation on nutrient transport pathways, and a better understanding of mechanisms will in turn, ultimately reveal the novel therapeutic strategies to treat the devastating diseases.
